# Hypoxia Enhances the Toxicity of Corexit EC9500A and Chemically Dispersed Southern Louisiana Sweet Crude Oil (MC-242) to Sheepshead Minnow (*Cyprinodon variegatus*) Larvae

**DOI:** 10.1371/journal.pone.0128939

**Published:** 2015-06-25

**Authors:** Subham Dasgupta, Irvin J. Huang, Anne E. McElroy

**Affiliations:** School of Marine and Atmospheric Sciences, Stony Brook University, Stony Brook, New York, United States of America; University of Tuscia, ITALY

## Abstract

Oil exploration and production activities are common in the northern Gulf of Mexico as well as many other coastal and near coastal areas worldwide. Seasonal hypoxia is also a common feature in the Northern Gulf, and many other coastal areas, which is likely to increase in severity and extent with continuing anthropogenic nutrient inputs. Hypoxia has well established physiological effects on many organisms, and it has been shown to enhance the toxicity of polycyclic aromatic hydrocarbons (persistent components of petroleum) in fish. The goal of this study was to examine the combined effects of hypoxia and exposure to contaminants associated with oil spills. We evaluated the effects of short term (48 hr) exposures to Corexit EC9500A, water accommodated fractions (WAF), and chemically enhanced water accommodated fractions (CEWAF) prepared from Southern Louisiana Sweet Crude Oil (MC 242) on survival of sheepshead minnow (*Cyprinodon variegatus*) larvae held under normoxic (ambient air) or hypoxic (2 mg/L O_2_) conditions. Results demonstrated that hypoxia significantly enhances mortality observed in response to Corexit or CEWAF solutions. In the latter case, significant interactions between the two stressors were also observed. Our data supports the need to further evaluate the combined stresses imparted by hypoxia and exposure to petroleum hydrocarbons and dispersants.

## Introduction

The 2010 Deepwater Horizon oil spill in the Gulf of Mexico was the largest accidental release of petroleum in U.S waters. Over 1.7 million gallons of oil were released, resulting in a large surface slick as well as a subsurface plume. In response to this, over 1.84 million gallons of dispersants were released at the surface and at depth in an attempt to mitigate the oil spill’s impact [1]. Petroleum and its associated products are common contaminants in marine systems well known to have the potential to negatively affect aquatic biota [2]. The widespread application of dispersants can influence this toxicity in complex ways [3]. The expansion of seasonal hypoxia is likely to provide another form of stress to organisms living in contaminated areas, particularly in the Gulf of Mexico but also in other coastal areas worldwide [4]. Although there is ample evidence of potential mechanisms by which co-exposure to hypoxia and oil could exacerbate the effects of their stressor alone, the combined effects of these stressors are not yet well appreciated [5].

Considering the intensity of oceanic oil extraction operations around the world, coastal areas will continue to be at risk from accidental oil releases. In some cases, these spills will superimpose themselves over expanding hypoxic zones. Large watersheds in agricultural or heavily populated areas, such as the Mississippi River drainage basin, act as a major source of nutrients to coastal waters [6]. Excess nutrients enhance the primary production of these areas, increasing phytoplankton and macroalgae biomass. Decaying plant matter fuels bacterial respiration, greatly increasing biological oxygen demand leading to hypoxic or anoxic conditions, particularly in benthic environments. Hypoxic events can trigger mass mortality of aerobic organisms or alter population fitness and have great potential for altering the ecosystems in which they occur [4]. Anthropogenic activities have already increased nutrient loading from large rivers and have served to exacerbate hypoxic events around the world [7]. The global trend of increasing hypoxic zones over the last couple of decades is likely to continue to grow in extent and severity [4].

A number of studies have assessed responses to hypoxia in marine organisms. Hypoxia is known to cause oxidative stress by causing an imbalance in oxidant/antioxidant production that can lead to a number of physiological effects [8]. For example, longjaw mudsucker (*Gillichthys mirabilis)* exposed to hypoxia in the laboratory displayed altered expression of genes involved with metabolism and locomotion, which may cause abnormal body functions [9]. Up-regulation of genes involved in stress and immune responses and down-regulation of genes involved in metabolism and growth have been reported for zebrafish (*Danio rerio*) exposed to hypoxia, indicating a shift from normal metabolism and development to stress acclimation [10]. Other studies with early life stage zebrafish have demonstrated hypoxia to be an environmental teratogen, strongly affecting fish development [11]. In recognition of the many field and laboratory assessments documenting the negative effects of hypoxia, the U.S. Environmental Protection Agency’s Ambient Water Quality Criteria for Dissolved Oxygen (seawater) set 2.3 mg/L as the lower limit for acute effects in juvenile and adult organisms experiencing either persistent or cyclic hypoxia [12].

Offshore oil exploration has long been an important industry in many coastal areas, including the Gulf of Mexico. Oil exposure has been shown to be toxic to many aquatic organisms with fish embryos and larvae being particularly sensitive to both lethal and sublethal effects [2]. Polycyclic aromatic hydrocarbons (PAHs) are considered major drivers of petroleum toxicity because of their persistence, cardiovascular effects and mutagenic and carcinogenic potential [13–15]. Research into the molecular pathways of response to both PAH and hypoxia shows evidence for interaction between the two processes. PAH metabolism and responses to hypoxia share a dimerization partner, the aryl hydrocarbon receptor nuclear translocator (ARNT), and may share other transcription factors [16]. Co-exposure to hypoxia has been shown to enhance the toxicity of PAHs in early and juvenile life stage fish [17–18]. However, the combined effects of oil exposure and hypoxia have received little attention to date.

Application of dispersants is one of the few tools available to mitigate that effects of oil spilled at sea. Dispersants are mixtures of a variety of surfactants and solvents, which reduce the interfacial tension between oil and water, creating smaller oil droplets that are entrained into the water column by wave energy [3]. Their use after a spill can effectively break up large oil slicks, minimizing oil deposition on shorelines and other sensitive coastal habitats. Although generally not considered very toxic themselves, studies with modern dispersants often conclude their presence enhances the toxicity of oil [19]. Laboratory studies exposing Atlantic herring (*Clupea harengus*) embryos to water accommodated fractions (WAF) or chemically enhanced water accommodated fractions (CEWAF) solutions found that CEWAF was almost 100 times more toxic than WAF. However, this enhanced toxicity was found to be due to dispersant increasing the aromatic hydrocarbon content of the test solutions [20]. Due to the ability of dispersants to increase exposure of aquatic organisms to petroleum compounds and the potential for interaction with hypoxia, dispersant use should be evaluated in a multiple stressor context.

The objective of our study was to examine the influence of hypoxia on contaminants associated with oil spills in coastal waters, evaluating survival in sheepshead minnow larvae after short term exposures to a commonly used oil spill dispersant, Corexit EC9500A (hereafter referred to as Corexit), as well as WAF and CEWAF solutions made from Southern Louisiana Sweet Crude Oil (MC-242) under normoxic and hypoxic conditions. Sheepshead minnows are common species found in the estuaries of the Gulf of Mexico [21] and have been shown to be sensitive to PAH exposure [22–23], making it a relevant model species in this study. Our results demonstrate that hypoxia significantly enhances toxicity, sometimes synergistically with contaminant exposure.

## Materials and Methods

### Sheepshead minnow rearing and production of larvae

Stony Brook University IACUC committee approved the care and use protocols for maintaining a breeding colony of sheepshead minnows Protocol #1470. This protocol acknowledges that embryos are being produced for early life stage (ELS) fish toxicity assessments including those specifically described in this study. After consultation with IACUC it was determined that specific approval for the ELS tests was not needed as all experiments were conducted on non-feeding yolk sac stage larvae. Adult sheepshead minnows, originally obtained from the Gulf Islands National Seashore in Pensacola FL (under permit number GUIS-2009-SI-0024 to Stephen Munch), were maintained in a recirculating artificial sea water (ASW, made with Instant Ocean, Blacksburg, VA) system with activated carbon filtration maintained on a 16:8 light/dark photo-cycle at 27–28^°^C, salinity 20–23 ppt. Embryos were held in ASW made in distilled water up to a salinity of 23 ±1 ppt at 28–29^°^C until hatch when they were transferred to treatment solutions made up in the same ASW. Larval exposures commenced at 1day post hatch (dph) and ran until 3dph. As the primary endpoint being assessed in this study was mortality, some larvae died as an effect of exposure to test solutions; larvae surviving the exposure were euthanized with 200 mg/L Tricaine-S.

### Source of chemicals

All chemicals used were of analytical grade or better and were obtained either from Sigma Aldrich (St. Louis, MO) or Fisher Scientific (Pittsburgh, PA), except when mentioned otherwise. Louisiana Sweet Crude Oil (MC-242) also known as Surrogate Oil was obtained from British Petroleum (Sample ID# 17449). Corexit was obtained as a gift from Columbia Analytical (Kelso WA).

### Preparation of test solutions

Concentration ranges tested were based on independent dilutions of stock solutions prepared fresh for each experiment. Reported concentrations for the Corexit stock solution are based on gravimetric determination. Analysis of representative samples of two of the major and persistent components in Corexit (dioctyl sodium sulfosuccinate and dipropylene glycol butyl ether) indicated initial dosing concentrations averaged 107±10% of those determined by gravimetric analysis. CEWAF (1g oil/L) and WAF (10 g oil/L) stock solutions were prepared following methods recommended by Adams et al. [20] with a Corexit:oil ratio of 1:10 for CEWAF. The solutions were stirred for 18 hrs with ~25% vortex, transferred to separatory funnels and allowed to settle for 6 hrs prior to removal of the WAF or CEWAF solutions. Subsequent dilutions were made in ASW. Preliminary range finding experiments were used to determine target loadings to be used in subsequent experiments (See S1 Table). Samples were stored in 50% ethanol for fluorescence spectroscopy. WAFs and CEWAFs were protected from direct light to minimize photo-oxidation, and all glassware used for the preparation of stock solutions was solvent rinsed with methanol and acetone prior to use. ASW was used as a negative control for all exposures.

### Measurement of oil in test solutions by fluorescence spectrometry

The actual concentrations of oil in our prepared WAF or CEWAF test solutions were estimated using fluorescence spectroscopy following methods described in [20] using a Hitachi 4500 fluorescence spectrometer (Pleasanton, CA) set at 265 nm excitation and 370 nm emission wavelengths with Southern Louisiana Sweet Crude Oil dissolved in 50:50 ethanol: ASW as a standard. Fluorescence at these wavelength pairs detects primarily 2–4 ring aromatic hydrocarbons [20]. All WAF and CEWAF loadings in this study are represented as their aromatic hydrocarbon concentrations.

### Sheepshead minnow larvae survival assays

All experiments were done in 0.5 ml flat bottom glass vials using 1 dph sheepshead minnow larvae. Each treatment had 15 replicate vials and each vial contained 1 larvae and 400 μL of test solution. During experiments vials were kept at room temperature at 23±1°C in ambient air or under hypoxic conditions in a sealed glove box with oxygen levels reduced to ~ 2mg/L by addition of purified N_2_ gas. Preliminary evaluation determined that equilibration between solutions in vials and ambient air in the glove box was complete within 2 hrs. Oxygen levels in the glove box were monitored using an OM-4 Oxygen Meter (Bedford, NH). Exposures lasted 48 hrs under 16:8 light: dark conditions with vials in oil exposures covered with aluminum foil to minimize photochemical reactions.

### Statistical analyses

All statistical analyses were done in R, a freely available programming package (http://www.r-project.org/). The binomial data obtained from the survival assays were fit into a generalized linear model and analyzed by a 2-way ANOVA using concentration and oxygen levels as the two factors. A p value of 0.05 was used to determine statistically significant change in survival.

## Results

Oil fluorescence studies showed that the aromatic hydrocarbon concentration of CEWAF was much higher than that of WAFs although initial oil loadings for WAFs were 10 times higher than CEWAFs (Table 1), demonstrating the well-known effectiveness of Corexit in dispersing oil [20].

**Table 1:** Nominal oil loadings and aromatic hydrocarbon concentrations of WAF and CEWAF samples.

Solution	Nominal loading (g/L)	Aromatic hydrocarbon concentration (mg/L)
**CEWAF**	**0.3**	**140**
	**0.5**	**200**
	**0.7**	**320**
	**0.9**	**450**
**WAF**	**2**	**3**
	**4**	**6**
	**6**	**10**
	**8**	**12**

**Fig 1:**
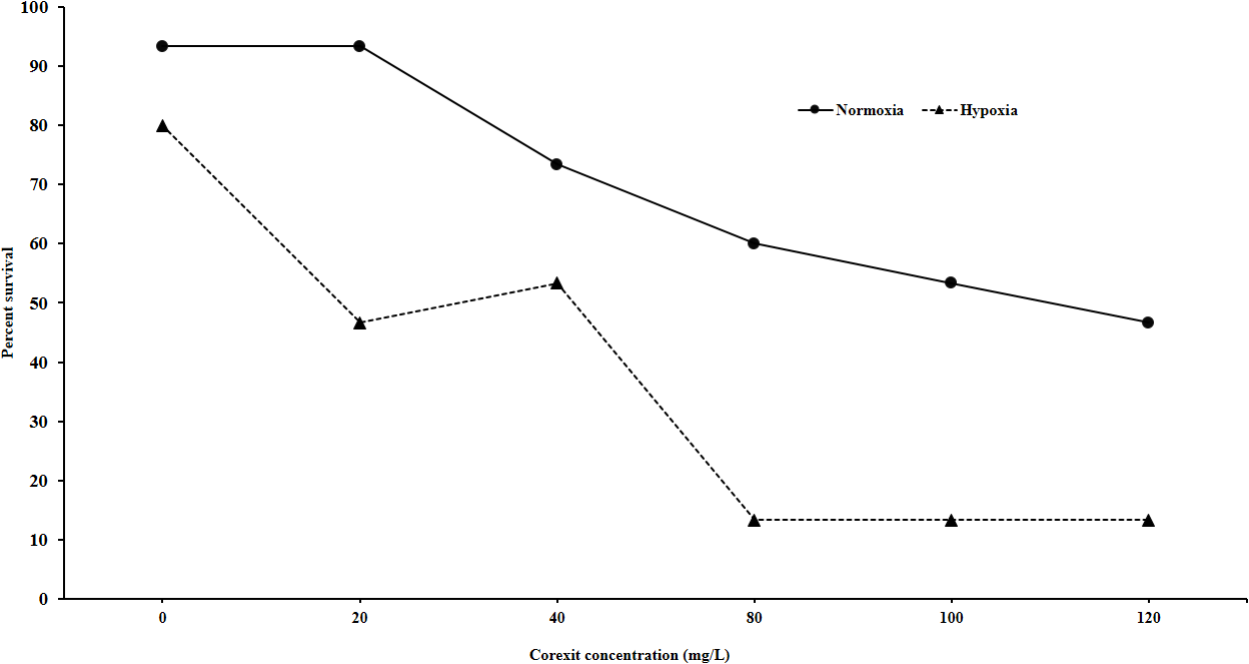
Effects of hypoxia on Corexit-induced acute toxicity in sheepshead minnow larvae. Statistical analysis by 2-way ANOVA demonstrates that both oxygen level and concentration have significant effects on survival (p<0.001) but their interaction is not significant (p = 0.56).

Exposure of sheepshead larvae to Corexit (0-200mg/L) showed that reduction in survival was concentration dependent and that hypoxia enhanced the toxicity (Fig 1). Survival was decreased by as much as 78% relative to effects under normoxic conditions. A two-way ANOVA demonstrated both oxygen and Corexit concentration caused statistically significant effects (p<0.001), but their interaction was not statistically significant (p = 0.56).

**Fig 2:**
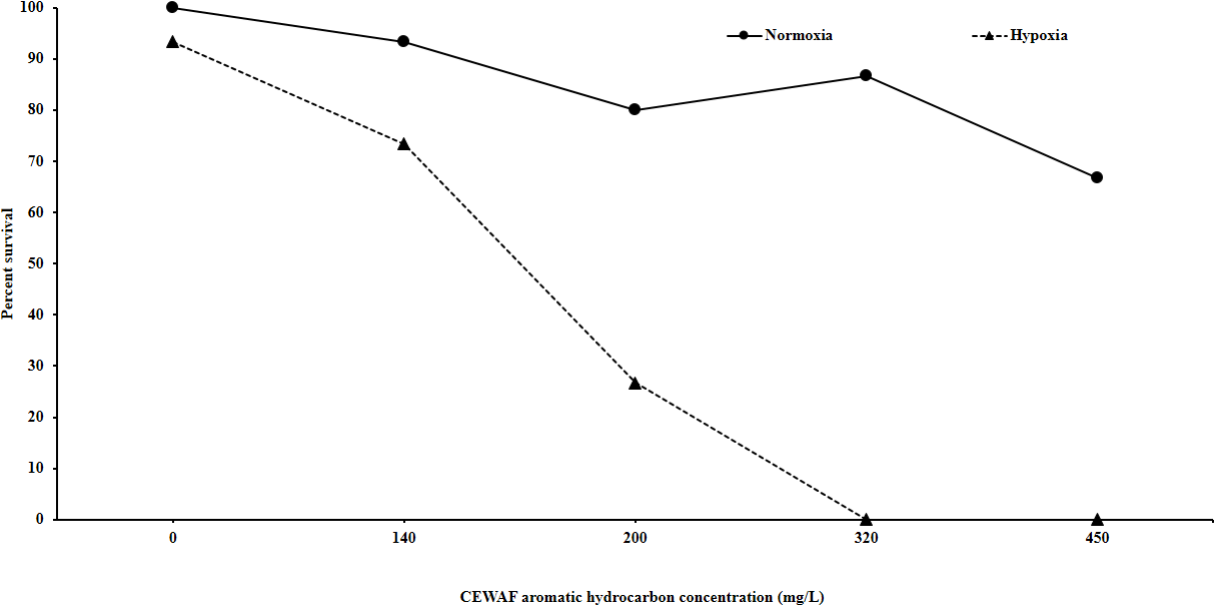
Effects of hypoxia on CEWAF-induced acute toxicity in sheepshead minnow larvae. CEWAF was prepared from Southern Louisiana Sweet Crude Oil and Corexit dispersant. Statistical analysis by 2-way ANOVA demonstrates that both oxygen level and concentration have significant effects on survival (p<0.001), and there are significant interactive effects between the treatments (p = 0.008).

More pronounced effects of hypoxia were observed in CEWAF exposures (oil loadings of 0–450 mg/L—Fig 2) where both oxygen and CEWAF concentration caused statistically significant reduction in survival individually (p<0.001), and a statistically significant interaction term was also identified (p = 0.008). In this experiment, survival was reduced by as much as 87% relative to effects under normoxic conditions.

**Fig 3:**
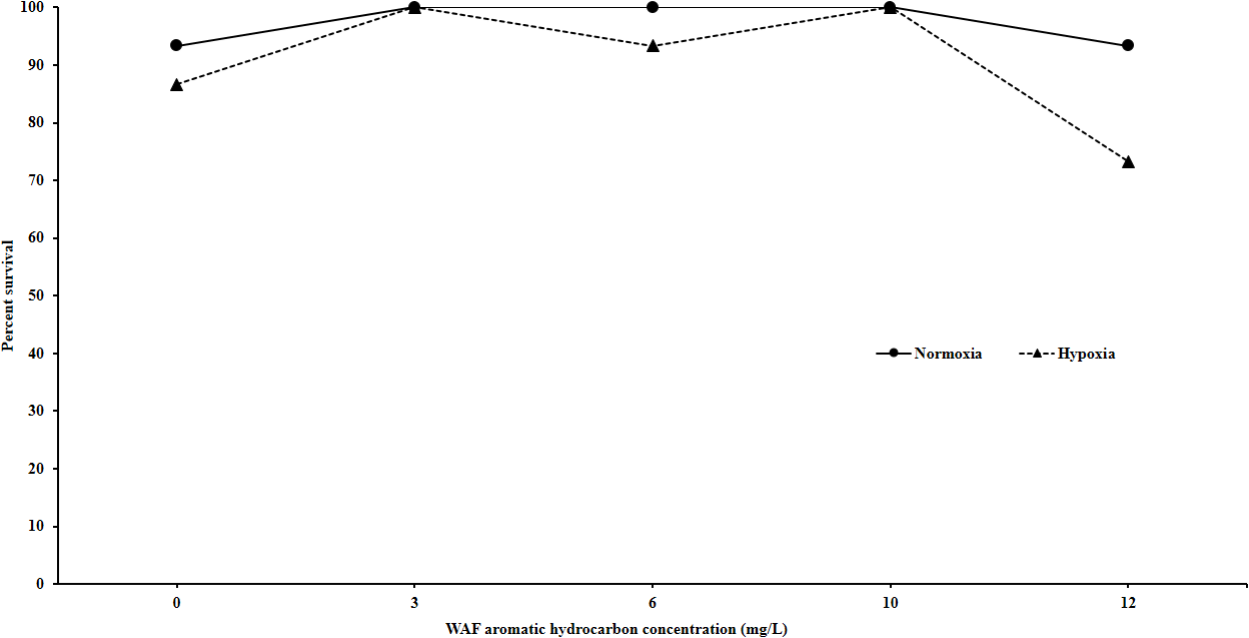
Effects of hypoxia on WAF-induced acute toxicity in sheepshead minnow larvae. WAF was prepared from Southern Louisiana Sweet Crude Oil. Statistical analysis failed to demonstrate any significant effects of either hypoxia or treatment or their interaction on larval survival (p = 0.33, p = 0.076, and p = 0.57 respectively).

In contrast, no significant effects of either concentration or hypoxic treatment were observed in exposures to WAF solutions (Fig 3). Looking at all three experiments, it is clear that there appears to be no reduction in survival in response to hypoxia alone (Figs 1–3).

## Discussion

Our studies show that hypoxia as a co-stressor significantly decreased survival of 1 dph sheepshead minnow larvae exposed to Corexit and CEWAFs, but not WAFs. The results obtained are consistent with preliminary experiments done previously to establish an appropriate dose response range, and are therefore considered robust. The influence of hypoxia was most pronounced in the CEWAF exposures where a synergistic effect of CEWAF and hypoxia was indicated by a statistically significant interaction term between these two treatments. In contrast to the combined effects of hypoxia and Corexit or CEWAF solutions, hypoxia alone did not appear to reduce survival in the short-term exposures evaluated in this experiment, indicating that sheepshead minnow larvae are able to withstand at least short term exposure to hypoxic conditions.

These results are consistent with previously published studies which show that hypoxia enhances the toxic effects of PAHs. Flemming and DiGuilio reported that zebrafish embryos exposed to a PAH mixture of 250 μg/L benzo[a]pyrene and 1000 μg/L 2-aminoanthracene showed significantly higher mortality under hypoxic (73%) as compared to normoxic (0%) conditions or hypoxic controls (2%) [24]. Co-exposure to hypoxia and PAH mixtures also induced higher incidence of developmental defects. One hypothesis for the interaction between PAH and hypoxia response is based on the involvement of ARNT in both PAH metabolism and hypoxia response. Hypoxia inducible factor-1α (HIF-1α) has been shown to competitively bind to ARNT, potentially reducing its availability to participate in aryl hydrocarbon receptor mediated induction of cytochrome P4501A (which metabolizes PAHs) [25]. Thus, hypoxia may lead to reduced PAH metabolism or lead to the production of more toxic metabolites through greater reliance on other metabolic pathways [17, 26]. Exposure to oil can also alter the structure of gills, affecting ion regulation and other gill functions like osmoregulation and gas exchange, potentially reducing the ability of the organisms to respond to hypoxic conditions in the environment [5]. We are aware of only one study examining the combined effects of oil and hypoxia on a marine organism. Negreiros et al. evaluated the effects of hypoxia and crude oil on DNA damage and gross pathology of the seahorse *Hippocampus reidi* [27], reporting significant elevations in DNA strand breaks in red blood cells after 8 hrs of exposure to either hypoxia, oil or the combination of both stressors. The highest level of effects observed resulted from the combined effects of oil and hypoxia and these results are generally in agreement with our study.

We are not aware of published studies on the influence of hypoxia on the toxicity of Corexit. In our study, hypoxia also enhanced the toxicity of Corexit, although no significant interactive affects were observed between Corexit exposure and hypoxia. Dispersants, hypoxia, and some aryl hydrocarbon agonists such as PAHs are known to exert oxidative stress in biological systems [8, 28–30]. In addition to the potentially additive effects brought on by oxidative stress, it is possible that the presence of dispersants in the CEWAF solution might contribute to the synergistic interaction observed. The nature or degree of this contribution is unknown and will require further study.

In contrast to our results with Corexit or CEWAF, no statistically significant decrease in survival was observed under either normoxic or hypoxic conditions in WAF exposures. Given the low concentration of aromatic hydrocarbons in the WAF solution (the highest WAF exposure was < 0.1 times the lowest CEWAF exposure), this was not surprising. It is interesting to note that while hypoxia significantly enhanced the toxicity of CEWAF, if was not sufficient to cause a statistically significant increase in WAF toxicity. Possibly if a higher energy WAF had been prepared resulting in a greater loading of petroleum hydrocarbons in the test solutions, the enhanced toxicity associated with hypoxic conditions would have been detectable.

In addition to aqueous exposures, many persistent petroleum hydrocarbons and some dispersant components become associated with particulate matter due to their hydrophobic nature and are deposited in marine sediments [2]. Recent reports have demonstrated significant deposition of petroleum hydrocarbons [31] and DOSS [32] in deep sediments of the Gulf of Mexico impacted by the Deep Water Horizon spill. Given the potential for sedimentary environments to also experience persistent or seasonal hypoxia, the interactive effects of dispersants, petroleum hydrocarbons and hypoxia need to be better understood to fully address potential risks, particularly to coastal benthic or demersal organisms.

Our study is one of the first to describe the combined effects of dispersants, dispersed oil, and hypoxia on early life stage fish. Future work will be needed to uncover the mechanistic foundation for these responses, other sublethal toxic endpoints resulting from the co-occurrence of these stressors, and the potential for longer termed effects due to early life stage exposure.

## Supporting Information

S1 Table Percent survival in 1dph sheepshead minnow larvae exposed to CEWAFs prepared from different oil loadings and an oil: dispersant ratio of 10:1. (DOCX)
