# Supplementary material for: Hypoxia Enhances the Toxicity of Corexit EC9500A and Chemically Dispersed Southern Louisiana Sweet Crude Oil (MC-242) to Sheepshead Minnow (Cyprinodon variegatus) Larvae
Source: PLoS One. 2015 Jun 25;10(6):e0128939. doi: 10.1371/journal.pone.0128939 (PMC4482444; doi:10.1371/journal.pone.0128939)
Supplement: S1 Table — (DOCX) [file pone.0128939.s001.docx]

**Table S1. Preliminary range finding experiments.**

Exposure concentrations for CEWAF treatments were based on a series of initial range-finding experiments to determine a range of concentrations where we could expect partial survival under normoxic conditions. Results of these experiments are provided in Table S1. All concentrations are based on nominal oil loadings. Our highest exposure range for WAF prepared from a solution of 8g/L oil did not show any significant mortality, hence the reported exposure range is the only range tested. For Corexit, exposure ranges were based on unpublished data from a separate study conducted in our laboratory.

| **Experiment (number of replicates)** | **CEWAF (g/L oil loading)** | **Percent survival** |
| --- | --- | --- |
| **Experiment 1 (8)** | 0.0090 | 88 |
|  | 0.090 | 88 |
|  | 0.90 | 88 |
| **Experiment 2 (8)** | 0.50 | 50 |
|  | 1.25 | 0.13 |
|  | 2.5 | 0 |
|  | 4.0 | 0 |
| **Experiment 3 (8)** | 0.25 | 100 |
|  | 0.50 | 100 |
|  | 1.0 | 0 |
|  | 2.0 | 0 |

**Table S1.** **Percent survival in 1dph sheepshead minnow larvae exposed to CEWAFs prepared from different oil loadings and an oil:dispersant ratio of 10:1.**
